# Use of Imaging Techniques to Illuminate Dynamics of Hematopoietic Stem Cells and Their Niches

**DOI:** 10.3389/fcell.2017.00062

**Published:** 2017-06-13

**Authors:** Takayuki Morikawa, Keiyo Takubo

**Affiliations:** Department of Stem Cell Biology, Research Institute, National Center for Global Health and MedicineTokyo, Japan

**Keywords:** hematopoietic stem cell, niche, imaging, bone marrow, hematopoiesis

## Abstract

Continuous generation of blood cells over an organism's lifetime is supported by hematopoietic stem/progenitor cells (HSPCs) capable of producing all hematopoietic cell subtypes. Adult mammalian HSPCs are localized to bone marrow and regulated by their neighboring microenvironment, or “niche.” Because interactions of HSPCs with their niches are highly dynamic and complex, the recent development of imaging technologies provides a powerful new tool to understand stem cell/niche biology. In this review, we discuss recent advances in our understanding of dynamic HSPC/niche interactions during development, homeostasis, disease states or aging with a focus on studies advanced by imaging analysis. We also summarize methods to visualize HSPCs and niche cells *in vivo*, including use of HSPC reporter mice and chemical probes. Findings emerging from these investigations could suggest novel therapies for diseases and aging.

## Introduction

In mammals, a lifetime supply of mature blood cells by a process known as hematopoiesis is maintained by differentiation and proliferation of hematopoietic stem/progenitor cells (HSPCs) in response to physiological or pathological stimuli. Removal of aging hematopoietic cells by phagocytes is a physiological stimulus for blood cell generation, while massive loss of mature blood cells due to infection, inflammation or bleeding functions as a pathological stimulus for hematopoiesis. Both types of stimuli alter gene expression and/or post-transcriptional events that prompt cell cycle activation or changes in cell fate decisions by hematopoietic stem cells (HSCs) to produce more fate-restricted progenitors. Those cells then produce mature blood cells to supply lost populations. Based on analysis of the hematopoietic system, which emerges from HSCs, one trillion blood cells are reportedly produced daily in an average human weighting 70 kg under physiological conditions (Ogawa, 1993). The detailed analysis of spatiotemporal regulation of hematopoiesis could foster development of novel therapies and diagnostics for infection, immunological disease, and hematological malignancies.

Use of imaging techniques has revealed that hematopoietic activities in both steady state and pathological conditions are dynamic and that their sequence is regulated spatiotemporally by interaction with the niche. Further development and application of imaging techniques, including *in vivo* HSC labeling, has revealed critical details relevant to the biology of the hematopoietic system (Kataoka et al., 2011; Chen et al., 2012; Koechlein et al., 2016; Sawai et al., 2016). Here, we review recent advances relevant to *in vivo* and *in vitro* imaging analysis of HSCs and their niches and discuss future directions.

## HSC visualization

### Labeling strategies useful for HSC tracking

Flow cytometry is commonly used to identify and purify HSCs in bone marrow. In this method, bone marrow cells stained by fluorophore-labeled antibodies that recognize HSC cell surface markers are sorted and injected into immunosuppressed mice. Consequently, donor HSCs engraft in bone marrow, enabling prospective identification and isolation of HSCs that exhibit self-renewal and multi-differentiation capacity *in vivo*. However, this method cannot provide spatial and temporal information relevant to HSC dynamics with the niche, an analysis that requires bone marrow dissection. The direct visualization of bone marrow is required to analyze HSCs in the context of the niche.

Microscopic analysis has helped define HSC niche structure (Table 1): briefly, confocal microscopy is used to scan bone marrow sections stained immunohistochemically and provides clear image at high speed (Joseph et al., 2013). Whereas it is hard to obtain images from deep part of tissue by using confocal microscopy, the light sheet microscopy allows us to visualize the deep portion of bone marrow (Chen et al., 2016; Greenbaum et al., 2017). Intravital deep imaging enabled by multi-photon microscopy has allowed analysis of cellular and oxygen dynamics in murine calvarial bone marrow.

**Table 1:** Listed are advantages and disadvantages of major options for imaging the HSC niche (Lieschke and Currie, 2007; Joseph et al., 2013).

**Equipment**	**Advantages**	**Disadvantages**	**Possible outcome**
Electron microscope	Very high resolution	Unsuitable for *in vivo* imaging	Ultrastructural features of HSC niche
Confocal microscope	High resolution High scan speed	Limited observing depths Photo-bleaching effect Phototoxic impact	Positional relationship between HSPC and niche cells
Multi-photon microscopy	Deeper observation depth Minimum photo-bleaching effect Lower phototoxicity	Limited scan speed Expense	Dynamics of HSPCs and niche in bone marrow
Light sheet microscopy	Excellent observation depth High scan speed Minimum photo-bleaching effect Lower phototoxicity	Unsuitable for tissue with strong light scattering property	Conformation of niche structure in whole bone marrow
**TARGETS**			
*In vitro*	Many tissues can be subjected to observation Numerous types of factors can be visualized	Physiological properties may not be revealed	Microstructure of HSC niche in long bone
*In vivo*	Biological responses can be observed	Limited observable regions	Pathophysiological phenomenon in the HSC niche
**DIMENSION**			
2D	Distance can be measured	Unsuitable for structural understanding of bone marrow	Distance between HSPC and niche cells
3D	Tissue geometry is easy to understand	Limited temporal resolution	Shape and alignment of HSPCs and niche cells
**SPECIES**			
Mouse	Various transgenic lines for HSPCs and niche cells are available	Poor tissue transparency	HSPC/niche interactions in bone marrow
Zebrafish	Higher optical clarity More rapid life cycle	Anatomical similarity to terrestrial mammals is limited	HSPC/niche interactions during development

Classically, labeling of HSPCs by fluorescent dyes, including carboxyfluorescein succinimidyl ester (CFSE), has been used to track transplanted HSPCs in bone marrow, and methods used to detect transplanted fluorophore-labeled HSPCs include flow cytometry, confocal microscopy, or multi-photon microscopy. Given that fluorophore-labeled cells lose fluorescence at each cell division (Weston and Parish, 1990; Lyons and Parish, 1994), fluorescence intensity also reflects the cell division history of transplanted cells over time (Takizawa et al., 2011). Insertion of intravital flexible microprobe into mouse femoral bone reveals that transplanted CFSE-labeled HSCs associate with vascularized structures in the femoral head (Lewandowski et al., 2010).

HSPC labeling requires HSPC isolation and incubation with dyes *ex vivo* prior to transplantation, and therefore this method allows analysis of only short-term dynamics after transplantation. Various transgenic reporter zebrafish and mice have been established to obtain spatial and temporal information relevant to normal dynamics of HSPCs by imaging analysis (Table 2). For example, promoter/enhancers of genes expressed primarily in murine HSCs (such as Evi1, Hoxb5, Pdzk1ip1, or Musashi2) are utilized to drive expression of fluorescent protein reporter genes (Kataoka et al., 2011; Chen et al., 2012; Koechlein et al., 2016; Sawai et al., 2016). Reporter mice enabling detection of HSCs and endothelial cells (ECs) have also been used to identify HSCs in bone marrow (Gazit et al., 2014; Acar et al., 2015). Although discrepancies in location between endogenous factors and reporter constructs occasionally occur, transgenic animals harboring reporters are powerful tools useful to visualize HSPCs in various hematopoietic organs, including bone marrow.

**Table 2:** Examples of key studies using reporter mice to detect HSPCs.

**Driver element**	**Reporter**	**Methods**			**Analysis**	**References**
Zebrafish CD41 (Tg)	GFP	Zebrafish	*In vivo*	Imaging Clonal fate mapping	Confocal microscopy Flow cytometry	Henninger et al., 2017
Zebrafish runx1 (Tg)	GFP mCherry	Zebrafish	*In vivo*	Imaging	Confocal microscopy Flow cytometry	Tamplin et al., 2015
Zebrafish runx1 (Tg)	GFP	Zebrafish	*In vivo*	Imaging	Confocal microscopy Flow cytometry	Hall et al., 2012
Zebrafish CD41 (Tg)	GFP	Zebrafish	*In vivo*	Imaging	Confocal microscopy	Kissa and Herbomel, 2010
Mouse Msi2 (KI)	eGFP	Mouse	*In vivo*	Imaging	Confocal microscopy	Koechlein et al., 2016
Mouse Hoxb5 (KI)	Tri-mCherry	Mouse	*In vivo In vitro*	Transplantation Tissue clearing	Flow cytometry Lightsheet microscopy	Chen et al., 2016
Mouse Pdzk1ip1 (Tg)	GFP	Mouse	*In vitro*	Doxycycline chase Transplantation	Flow cytometry	Sawai et al., 2016
Human CD34-tTA (Tg)	H2B-GFP	Mouse	*In vivo*	Doxycycline chase Transplantation	Flow cytometry	Bernitz et al., 2016
Mouse α-catulin (KI)	GFP	Mouse	*In vitro*	Tissue clearing Immunostaining	Confocal microscopy Multi-photon microscopy	Acar et al., 2015
Mouse Fdg5 (KI)	mCherry	Mouse	*In vivo*	Transplantation	Flow cytometry	Gazit et al., 2014
Mouse Vwf (Tg)	eGFP	Mouse	*In vivo*	Transplantation	Flow cytometry	Sanjuan-Pla et al., 2013
Mouse Scl-tTA (Tg)	H2B-GFP	Mouse	*In vivo In vitro*	Doxycycline chase Immunostaining	Flow cytometry Confocal microscopy	Sugimura et al., 2012
Mouse Evi1 (KI)	GFP	Mouse	*In vivo*	Transplantation	Flow cytometry	Kataoka et al., 2011
Mouse Ly6a (Tg)	GFP	Mouse	*Ex vivo*	Imaging	Confocal microscopy	Boisset et al., 2010
Mouse Scl-tTA (Tg)	H2B-GFP	Mouse	*In vivo*	Doxycycline chase	Flow cytometry	Wilson et al., 2008

*Tg, Transgenic; KI, Knock-in; tTA, Tetracycline-controlled transactivator protein*.

### Imaging of HSC movement and location

**Figure 1:**
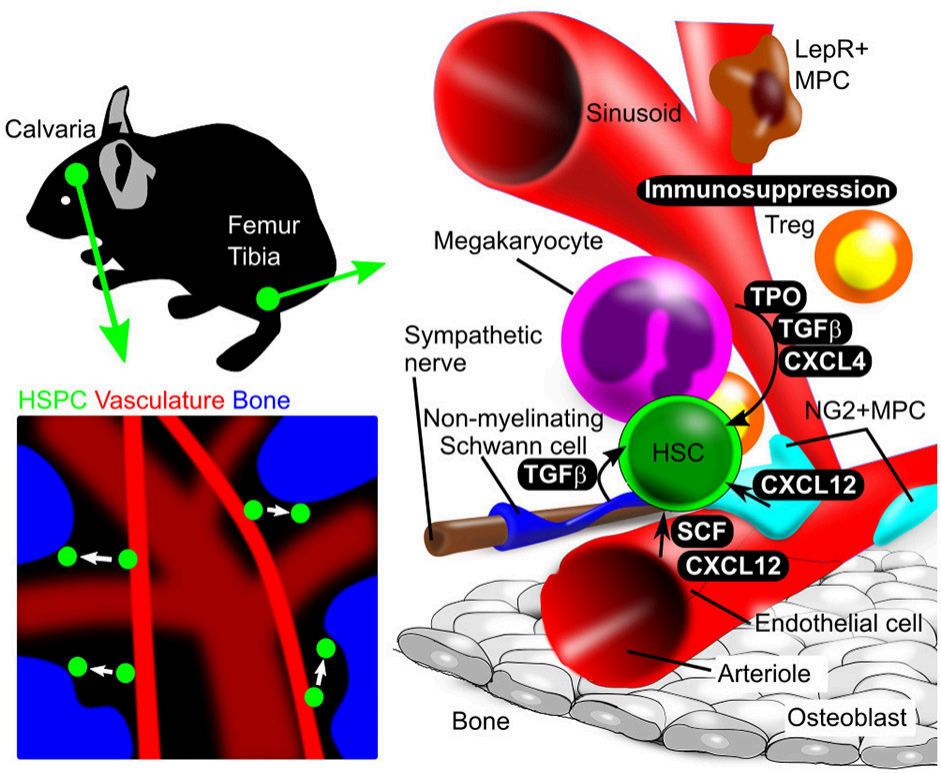
Illustration of *in vivo* and *in vitro* bone marrow imaging. (Upper left panel) Calvarial bone marrow subjected to *in vivo* imaging. Use of reporter mice and *in vivo* staining allows HSPC detection in calvarial bone marrow. (Lower left panel) Intravenous injection of fluorescent dye (red) and second harmonics generation (blue), respectively, identify blood vessels and bone. HSPC behavior is analyzed using a chemical or genetic fluorescent reporter (green). (Right panel) Schematic showing femoral and tibial bone marrow, including HSPCs and niche cells, as revealed by immunostaining. Niche components and their spatial relationships can be observed by imaging analysis.

Transgenic reporter mice have made it possible to detect HSCs and track their fate *in vitro* and *in vivo* based on fluorescence imaging. For instance, mice created using knock-in of a reporter driven by the RNA-binding protein Musashi2 (Msi2) enabled confocal laser scanning microscopy analysis of HSPC movement in calvarial bone marrow (Koechlein et al., 2016); that study revealed that HSPCs residing near vessels migrate toward close proximity to endosteum (Figure 1).

Also, GFP knock-in into the α-catulin gene, which is dominantly expressed in HSCs, allowed detection of HSCs in the niche (Acar et al., 2015). Use of these mice combined with techniques to clear bone and bone marrow has provided microscopic evidence that the HSC niche is perisinusoidal in bone marrow (Acar et al., 2015).

### Tracking of HSC division

In addition to the HSC-specific promoter/enhancer-based labeling techniques, the non-dividing phenotype of highly primitive HSCs has been exploited to analyze and purify HSCs. Retaining of 5-bromo-2-deoxyuridine (BrdU) by long-term quiescent HSCs serves as a way to detect this cell type (Wilson et al., 2008). However, non-dividing cells that retain the BrdU label can be identified only after fixation, which kills cells, and this approach is not suitable to isolate living, quiescent HSCs for further analysis.

To resolve this difficulty, a tetracycline (Tet)-inducible expression system employing a histone H2B/fluorescent protein fusion gene was developed (Wilson et al., 2008; Foudi et al., 2009; Sugimura et al., 2012; Bernitz et al., 2016; Säwén et al., 2016). This system is based on the idea that mature hematopoietic cells and HSPCs express the basic helix-loop-helix transcription factor stem cell leukemia (Scl, also known as Tal1), a factor that regulates embryonic and adult hematopoiesis by HSC production and maintenance (Robb et al., 1995; Shivdasani et al., 1995; Mikkola et al., 2003).

A knock-in mouse line harboring the tetracycline transactivator (tTA) under control of endogenous Scl could mark Ter119^+^ erythroid cells, Gr-1^+^ granulocytes, CD41^+^ megakaryocytes and lineage marker (Lin)-negative c-Kit^+^ HSPCs (Bockamp et al., 2006). This line is then crossed to a transgenic line expressing the histone H2B-GFP fusion protein under control of a tetracycline-responsive regulatory element (TRE). In Scl-tTA::TRE-H2B-GFP double heterozygous mice, greater than 80% of HSC/MPPs express GFP at high levels. In one analysis, after 70 days of doxycycline chase, Lin^−^ GFP^bright^ cells were highly enriched for non-cycling HSCs (Wilson et al., 2008). Using this system, non-dividing GFP^bright^ HSCs and niche cells were visualized by confocal microscopy, and HSCs were seen in contact with N-cadherin-positive osteoblasts and these osteoblasts activate non-canonical Wnt signaling in the HSC niche (Sugimura et al., 2012). Another seminal study using the hCD34-tTA::TRE-H2B-GFP line showed that HSCs reach a state of complete dormancy after four self-renewal events (Bernitz et al., 2016). The identity of factors that regulate spatiotemporal dynamics of HSC division over this time is a topic for future investigation.

As noted above, while simultaneous identification of multiple cell types remains imperfect due to the limited number of fluorescent dyes applicable to a single experiment, the emergence of novel imaging technologies has facilitated analysis of HSPC movement and molecular interactions in the niche.

## Use of imaging technologies to analyze the HSC niche during development

Imaging technology can reveal spatiotemporal cellular interactions and dynamics in the HSC niche. In particular, transgenic animal lines expressing fluorescent proteins in niche cells enable visualization of HSPC interaction with the hematopoietic microenvironment (Table 3). Zebrafish are now a powerful model in which to define mechanisms relevant to hematopoiesis and characterize HSC interactions with the microenvironment that govern development (Lieschke and Currie, 2007). In zebrafish, hematopoietic cell lineages are derived from posterior lateral mesoderm (PLM) cells, and Notch signaling between PLM cells and their scaffold, somitic cells, is required for hematopoietic development (Kobayashi et al., 2014). Using two-dimensional (2D) time-lapse live imaging of zebrafish embryo has revealed that HSCs are derived directly from aortic endothelium during development (Bertrand et al., 2010; Kissa and Herbomel, 2010). HSPCs then enter the bloodstream and arrive at the endothelial network in the ventral region of the embryo called the caudal hematopoietic tissue (CHT). HSPCs subsequently attach to the endothelium and remodel the CHT vascular plexus to form a surrounding pocket serving as a site for HSPC division (Tamplin et al., 2015).

**Table 3:** Examples of key studies using reporter mice to detect niche cells by genetically expressing fluorescent protein.

**Driver element**	**Reporter**	**Target cell**	**Model**		**Analysis**	**References**
Zebrafish kdrl (Tg) Zebrafish cxcl12a (Tg)	GFP, mCherry DsRed2	ECs Stromal cells	Zebrafish	*In vivo*	Confocal microscopy Flow cytometry	Tamplin et al., 2015
Zebrafish kdrl (Tg) Zebrafish fli1 (Tg)	GFP, DsRed mCherry	ECs ECs	Zebrafish	*In vivo In vitro*	Confocal microscopy Flow cytometry	Kobayashi et al., 2014
Zebrafish kdrl (Tg)	mCherry	ECs	Zebrafish	*In vivo*	Confocal microscopy Flow cytometry	Hall et al., 2012
Zebrafish kdrl (Tg)	GFP, dTomato	ECs	Zebrafish	*In vivo*	Confocal microscopy	Kissa and Herbomel, 2010
Zebrafish kdrl (Tg)	mCherry	ECs	Zebrafish	*In vivo*	Confocal microscopy Flow cytometry	Bertrand et al., 2010
Mouse Efnb2 (Tg) Mouse Flk1 (Tg)	GFP GFP	ECs ECs	Mouse	*In vivo*	Multi-photon microscopy	Bixel et al., 2017
Mouse Sca-1 (Tg) Rat nestin (Tg)	EGFP EGFP	ECs ECs	Mouse	*In vivo In vitro*	Confocal microscopy Multi-photon microscopy	Itkin et al., 2016
Rat nestin (Tg)	GFP	MSCs	Mouse	*In vivo*	Multi-photon microscopy	Spencer et al., 2014
Mouse Cxcl12 (KI)	GFP	MPCs	Mouse	*In vitro*	Confocal microscopy	Greenbaum et al., 2013
Rat nestin (Tg)	GFP	MSCs	Mouse	*In vitro*	Confocal microscopy	Kunisaki et al., 2013
Mouse CxclL12 (KI) Mouse Scf (KI) Rat Col2.3 (Tg)	DsRed GFP GFP	EC PVSCs Perivascular cells Osteoblasts	Mouse	*In vitro*	Confocal microscopy	Ding and Morrison, 2013
Mouse Scf (KI) Rat nestin (Tg) Rat nestin-cre (Tg) Rat Col2.3-cre (Tg) Mouse Lepr-cre (KI)	GFP GFP Cherry loxP-EYFP loxP-EYFP loxP-EYFP	Perivascular cells PVSCs PVSCs Osteoblasts PVSCs	Mouse	*In vitro*	Confocal microscopy	Ding et al., 2012
Mouse Foxp3 (KI)	GFP	T_reg_s	Mouse	*In vivo*	Multi-photon microscopy	Fujisaki et al., 2011
Mouse Cxcl12 (KI)	GFP	CAR cells	Mouse	*In vitro*	Confocal microscopy	Omatsu et al., 2010
Rat Col2.3 (Tg)	GFP	Osteoblasts	Mouse	*In vivo*	Multi-photon microscopy	Lo Celso et al., 2009
Mouse Vegfr2 (KI) Rat Col2.3 (Tg)	GFP GFP	Sinusoidal ECs Osteoblasts	Mouse	*In vitro*	Confocal microscopy	Hooper et al., 2009

*Many of these lines have been used for imaging studies. Tg, Transgenic; KI, Knock-in; EC, Endothelial cells; PVSCs, Perivascular stromal cells; CAR cells, CXCL12-abundant reticular cells*.

Zebrafish models have also been useful to define embryonic HSC niche function. For example, nitric oxide production in the aorta-gonad-mesonephros (AGM) region is critical for a larval hematopoietic response to bacterial infection, as shown by studies using three-dimensional (3D) confocal live imaging (Hall et al., 2012). Since zebrafish embryos are relatively easy to manipulate, some have employed clonal mapping using multi-color genetic labeling and reported evidence suggesting that a limited number of HSC clones contributes to life-long hematopoiesis (Henninger et al., 2017). While wild-type zebrafish embryos are of high clarity, a transgenic line is now available with a body transparent enough for imaging analysis in adult fish (White et al., 2008).

In mammals, bone marrow HSCs are derived from embryonic hemogenic ECs in AGM. Fetal HSCs from AGM migrate to fetal liver (FL) or spleen and then expand their number. Analysis of Ly6a (Sca-1)-GFP transgenic mice, in which HSPCs are GFP-positive (Ma et al., 2002), combined with 3D confocal microscopy, has revealed the precise timing of HSC emerging can be visualized at the embryonic aortic endothelium (Boisset et al., 2010). Confocal microscopy-based 3D imaging of Ly6a-GFP embryos has revealed that HSPCs from FL interact with ECs (Tamplin et al., 2015). By improving sample preparation and imaging technology, longer time-lapse imaging of developmental stages will provide a more complete picture of HSC migration between organs.

Other imaging analysis has suggested that portal vessel-associated pericytes serve as critical HSC niche components in mouse FL (Khan et al., 2016). Specifically, in mice at birth, portal vessels change from a Neuropilin-1^+^Ephrin-B2^+^ artery to EphB4^+^ vein phenotype, resulting in pericyte loss and HSC release from FL. Perivascular lodgment of HSPCs induces active remodeling of the perivascular niche to promote HSPC expansion and maintenance in FL during development.

Post-natal hematopoiesis in mammals occurs mainly in bone marrow. Essential processes of bone development and ossification precede bone marrow development and begin embryonically. In the case of long bones, mineralization of cartilage is followed by blood vessel invasion of the central region of that tissue. Blood then perfuses bones, and actively dividing HSPCs arrive as early as E16.5 in mice, as revealed by 2D immunohistochemical analysis (Coskun et al., 2014). A recent study using *in vitro* imaging system reported that these HSPCs in fetal bone marrow switch from actively-dividing to quiescent, a transition mediated by osteoblast activity, as loss of osteolineage cells in *Osx*^−/−^ mice perturbs induction of HSPC quiescence (Coskun et al., 2014). Another study reports active division of murine HSCs in bone marrow until 3 weeks of age, but after 4 weeks HSCs stop dividing and become quiescent (Bowie et al., 2006). However, molecular and environmental cues that induce these phenotypic changes remain unclear.

## The adult HSC niche

### Structural and regional analysis of the adult HSC niche

Imaging analysis has demonstrated complex interactions between HSC and niche cells, as illustrated in Figure 1. In adult mouse bone marrow, the perivascular region is the major HSC niche and is composed of various cell types that function in HSC maintenance. To understand how HSC and various niche cells interact, it is crucial to know the histological structure and properties of bone marrow including vasculature components.

The types of blood vessels in bone marrow are described as follows. Arterial blood flow in bone marrow is mainly supplied by nutrient vessels that penetrate cortical bone. These vessels merge and then form the central artery of bone marrow. Arterioles branch from the central artery toward cortical bone and anastomose with the sinusoid. Transition zone vessels connect arterioles and sinusoidal vessels. Sinusoidal vessels then connect with the central vein, and blood flows from bone marrow through the nutrient vein (Li et al., 2009; Acar et al., 2015; Morikawa and Takubo, 2016). These vessels are classified by morphological or cellular characteristics revealed by imaging analysis.

Based on imaging analyses of bone marrow, both arteriolar and sinusoidal regions serve as HSC niches (Nombela-Arrieta et al., 2013). Functionally, arteriolar niche cells promote HSC quiescence and sinusoids represent a proliferative HSC niche (Kunisaki et al., 2013). *In vivo* imaging is now an essential not only to track cell movement but to obtain information relevant to blood flow and vascular permeability in bone marrow. Sinusoid exhibits higher vascular permeability than do arteries or arterioles, a property important for bidirectional trafficking of HSCs and differentiated cells between bone marrow and the circulation (Itkin et al., 2016). 3D vascular structural analysis and blood flow measurement using multi-photon laser microscopy suggest that sinusoidal blood flow and shear stress are lower than that seen in the arteriole (Bixel et al., 2017). This study shows that blood flow profiles modulate HSPC homing in the bone marrow vasculature and employs calvaria and femur for *in vivo* imaging and FACS analysis, respectively. Since it is known that hematopoiesis continues in flat bone predominantly in aged human, hematological differences exhibited by these bones are particular interest in future studies. Because the impact of anesthesia or surgical stress on hematopoiesis remains unclear, it is important to carefully interpret results from intravital imaging analysis.

Imaging in mouse has also identified a function of the endosteal region as a regulatory environment for HSCs. For example, *ex vivo* imaging of mouse bone reveals that engrafting HSCs are maintained in the endosteal region after irradiation (Xie et al., 2009). Furthermore, *in vivo* imaging shows that transplanted HSCs dive into close proximity to endosteum (Lo Celso et al., 2009), supporting the idea that the latter functions in HSC homing to damaged bone marrow.

### Interaction between niche cells and HSPCs in adult bone marrow

#### Endothelial cells

Bone marrow endothelium expresses the adhesion molecule E-selectin, playing role in the homing and engraftment of circulating HSPCs (Hidalgo et al., 2002; Katayama et al., 2003). Imaging techniques provides evidence that perisinusoidal HSC proliferation is stimulated by cellular interactions with E-selectin expressed on ECs (Winkler et al., 2012). Sinusoidal ECs also express vascular endothelial cell growth factor (VEGF) receptor 2, and VEGF signaling is required to reconstitute hematopoiesis and maintain HSCs after myeloablation (Hooper et al., 2009). Moreover, Notch ligand secreted by sinusoidal ECs promotes HSC proliferation (Butler et al., 2010).

Confocal microscopy of bone marrow from cytokine stem cell factor (Scf)-GFP knock-in mice revealed that ECs, which form the inner lumen of blood vessels, function in HSC maintenance by producing SCF (Ding et al., 2012).

#### Mesenchymal stromal cells

Mesenchymal Stromal cells (MSCs), which are associated with sinusoidal ECs, have been proposed as niche cells, as they produce factors important to maintain HSCs, such as SCF and CXCL12 (Omatsu et al., 2010; Ding and Morrison, 2013; Greenbaum et al., 2013). Mice engineered to harbor fluorescent reporters at the Scf or Cxcl12 loci provide support that MSCs highly express both genes and are required for HSC the maintenance in bone marrow. The application of tissue clearing methods to bone analysis has increased light transmission of tissue harboring fluorescent protein tags. Tissue clearing and whole bone marrow imaging by using light sheet microscopy of α-catulin-GFP mice demonstrates that in perisinusoidal regions, HSCs reside primarily with MSCs, which highly express the leptin receptor and Cxcl12 (Acar et al., 2015). In support of this finding, others have applied a tissue clearing method to bone marrow plugs of Hoxb5-Tri-mCherry mice, in which HSCs are specifically marked (Chen et al., 2016). In this analysis, Hoxb5^+^ HSCs are localized to the perivascular localization of bone marrow. Most of these HSCs are quiescent (Chen et al., 2016). Additional advances in tissue clearing techniques in mice now enable whole body imaging (Tainaka et al., 2014). These types of methodologies could allow analysis of HSPC distribution throughout the entire body.

Analysis using Nestin-GFP transgenic mice indicates that arterioles are associated with Nestin-GFP^bright^ perivascular stromal cells (Kunisaki et al., 2013). These cells have MSC properties *ex vivo*, highly express the pericyte marker NG2, and reside close to HSCs. Analysis of Nestin-GFP transgenic mice also shows that Nestin-GFP^dim^ cells associate with sinusoids (Kunisaki et al., 2013). Nestin-GFP^bright^ cells are more quiescent than Nestin-GFP^dim^ cells and highly express HSC niche factors. The periarteriolar niche may maintain HSCs in a more primitive state than those in the sinusoidal niche (Kunisaki et al., 2013).

#### Neurons and non-myelinating schwann cells

Immunohistochemical analysis shows that the periarteriolar niche, which harbors Nestin-GFP^bright^, cells, is innervated by sympathetic neurons (Méndez-Ferrer et al., 2008). Bone marrow sympathetic nerves release noradrenaline from terminals, an activity that reduces Cxcl12 expression in bone marrow stroma cells. As a result, sympathetic signaling activated by G-CSF promotes HSC release from the niche (Katayama et al., 2006; Méndez-Ferrer et al., 2008). The periarteriolar sympathetic nerve fibers are ensheathed by non-myelinating Schwann cells that activate a latent form TGF-β to maintain HSC quiescence (Yamazaki et al., 2011). 2D confocal imaging of bone marrow reveals that non-myelinating Schwann cells colocalize with HSCs and run parallel to arterioles and sympathetic nerves (Yamazaki et al., 2011; Itkin et al., 2016). These observations support the idea that the periarteriolar region forms a neurovascular-stromal unit that regulates HSC dynamics *in vivo*. Also, intravital imaging of the steps of that migration of G-CSF-stimulated HSPC mobilization from the niche is an area for further investigation.

#### Hematopoietic cells

In addition to mesenchymal lineage cells, hematopoietic cells function as HSC niche cells. While platelet production is a major function of megakaryocytes, they also produce niche factors, among them, Cxcl4, TGF-β, and thrombopoietin, in bone marrow. Confocal microscopy, whole-mount imaging and computational modeling suggest that megakaryocytes and HSCs co-localize (Bruns et al., 2014; Nakamura-Ishizu et al., 2014; Zhao et al., 2014).

Macrophages are critical for G-CSF-induced mobilization of HSCs and are considered a niche cell (Winkler et al., 2010; Chow et al., 2011). Confocal microscopic analysis reveals that macrophages reside in the vicinity of Nestin^+^ MSC niche cells, and crosstalk between these two cell types enhances HSC retention in the niche.

Regulatory T (T_reg_) cells suppress immune responses. Survival time of transplanted allogenic HSPCs in T_reg_ cell-depleted mice is shorter than that seen in intact mice as revealed by analysis of FoxP3-GFP reporter mice (Fujisaki et al., 2011). T_reg_ cells suppress immune responses at the HSC niche. *In vivo* imaging analysis using multi-photon microscopy also reveals spatial interactions between T_reg_ cells colocalizing with HSPCs (Fujisaki et al., 2011).

#### Non-cellular elements

Non-cellular elements also serve as HSC niche factors. Studies using computer simulations of pO_2_ distribution suggest that the hematopoietic compartment is relatively hypoxic (Chow et al., 2001), a condition that maintains HSCs by various mechanisms, including lowering levels of reactive oxygen species (ROS). Imaging has been used to assess the relationship between hypoxia and HSCs stemness. Imaging analysis using oxygen-sensing chemical probes now provides better understanding of molecular oxygen distribution in bone marrow. When incorporated into hypoxic tissues, pimonidazole, a hypoxia probe, can be detected by immunohistochemistry or flow cytometry with anti-pimonidazole antibodies. Using this technique, the HSPCs in bone marrow were found to be hypoxic (Takubo et al., 2010; Nombela-Arrieta et al., 2013).

Improved tissue clearing techniques combined with 3D imaging of thick bone marrow sections confirm that the hypoxic property of HSPCs is independent of their distance from the vasculature (Nombela-Arrieta et al., 2013). HSCs utilize the cellular hypoxia-response system to maintain quiescence and glycolytic metabolic properties (Takubo et al., 2010, 2013). Direct analysis of the bone marrow niche using a phosphorescence lifetime-based O_2_ sensing technique and intravital microscopy suggests that (i) bone marrow extracellular space is generally hypoxic and (ii) pO_2_ in the periosteum region, where arterioles reside, is higher than in the peri-sinusoidal region located far from the endosteum (Spencer et al., 2014). This study provided local pO_2_ information at different regions of bone marrow. Additional dynamic analysis of 2D/3D oxygen distribution in bone marrow and other organs will be required to fully understand how hypoxia maintains stemness of HSC.

## Use of imaging to analyze leukemia, infection, and age-related events in the HSC niche

*In vivo* imaging of bone marrow using a custom-built fluorescence confocal/multiphoton microscope revealed that pre-B-cell acute lymphoblastic leukemia (ALL) cells preferentially home to bone marrow vessels that express the adhesion molecule E-selectin and Cxcl12 (Sipkins et al., 2005). ALL cells also locally metastasize to Cxcl12-expressing vascular niche cells (Colmone et al., 2008). ALL cells also alter niche cell properties, decrease Cxcl12 production and induce SCF overexpression in bone marrow. *In vivo* time-lapse imaging of the T-ALL niche also reveals that T-ALL cells directly induce osteoblast shrinking and blebbing (Hawkins et al., 2016). Acute myelogenous leukemia and myeloproliferative neoplasms remodel the bone marrow microenvironment by disrupting niche cells, such as MSCs, neurons and Schwann cells (Arranz et al., 2014; Hanoun et al., 2014). As part of the host defense system, immune cells are consumed during infection, activating hematopoietic stem cells to supply blood cells (King and Goodell, 2011). Toll-like receptors and interferon receptors on HSPCs sense infection stress and activate a myeloid differentiation pathway called “emergency myelopoiesis” (Nagai et al., 2006). HSPCs also directly recognize the bacterial product bis-(3′-5′)-cyclic dimeric guanosine monophosphate (c-di-GMP) through the innate immune sensor STING. Activation of the c-di-GMP/STING pathway mobilizes HSPCs to peripheral blood (Kobayashi et al., 2015). c-di-GMP also suppresses expression of niche factors (namely, Cxcl12, SCF and Angiopoietin-1) in various non-hematopoietic niche cells. c-di-GMP induces expansion of the sinusoidal area of bone marrow, as revealed by 2D immunohistochemical analysis. Furthermore, *in vivo* time-lapse imaging during acute infection shows that HSC motility is more significantly activated after infection than in steady state (Rashidi et al., 2014). Infectious stress induces HSPC niche remodeling and facilitates HSPC mobilization.

Physiological aging also changes properties of the HSPC niche. Confocal imaging techniques have revealed that bone marrow arteries covered with the α-smooth muscle actin^+^ cells decrease in number and become more permeable with aging in mice (Kusumbe et al., 2016). Imaging analysis of animal models of hematological disease or aging will provide pathophysiological insights with potential therapeutic application.

## Conclusion

The dynamics of hematopoiesis are tightly regulated by HSPCs and their niches within the bone marrow. Imaging techniques provide novel methods to define spatiotemporal regulation of complex multicellular microenvironments like bone marrow that every year we know more and more (Joseph et al., 2013). Although various methodological and technological hurdles remain, use of diverse techniques brings increasing insight into HSC interaction with niche cells and reveals how hematopoietic homeostasis is achieved in a dynamic manner.

Here, we have provided examples of imaging-based investigation of various hematopoietic activities, including developmental, physiological and pathological conditions and aging. Studies discussed here focus not only on stem cell location but on properties of the niche environment, such as local oxygen conditions. We anticipate that visualization of HSC cellular status in the niche will define additional mechanisms underlying hematopoiesis and leukemogenesis and potentially suggest novel therapies for blood cell diseases. Achieving this aim will require development of novel chemical and genetic probes of the cell cycle, metabolism, and signaling status and application of those methods to HSPC biology.

## Author contributions

TM and KT wrote the manuscript; and KT conceived and supervised the project.

### Conflict of interest statement

The authors declare that the research was conducted in the absence of any commercial or financial relationships that could be construed as a potential conflict of interest.
